# Novel Reporter Alleles of GSK-3α and GSK-3β

**DOI:** 10.1371/journal.pone.0050422

**Published:** 2012-11-21

**Authors:** William B. Barrell, Heather L. Szabo-Rogers, Karen J. Liu

**Affiliations:** Department of Craniofacial Development and Stem Cell Biology, King’s College London, London, United Kingdom; Rutgers University, United States of America

## Abstract

Glycogen Synthase Kinase 3 (GSK-3) is a key player in development, physiology and disease. Because of this, GSK-3 inhibitors are increasingly being explored for a variety of applications. In addition most analyses focus on GSK-3β and overlook the closely related protein GSK-3α. Here, we describe novel GSK-3α and GSK-3β mouse alleles that allow us to visualise expression of their respective mRNAs by tracking β-galactosidase activity. We used these new *lacZ* alleles to compare expression in the palate and cranial sutures and found that there was indeed differential expression. Furthermore, both are loss of function alleles and can be used to generate homozygous mutant mice; in addition, excision of the *lacZ* cassette from GSK-3α creates a Cre-dependent tissue-specific knockout. As expected, GSK3α mutants were viable, while GSK3β mutants died after birth with a complete cleft palate. We also assessed the GSK-3α mutants for cranial and sternal phenotypes and found that they were essentially normal. Finally, we observed gestational lethality in compound GSK-3β^−/−^; GSK3α^+/−^ mutants, suggesting that GSK-3 dosage is critical during embryonic development.

## Introduction

Glycogen Synthase Kinase 3 (GSK-3) is a serine/threonine kinase which was first discovered as a regulator of glycogen biosynthesis [1]. Since then, GSK-3 has been shown to phosphorylate many other substrates. One example is β-catenin, an intracellular signalling molecule required in the canonical Wnt signalling pathway. GSK-3 also acts as a key regulator in a number of developmental signalling pathways, including Hedgehog, transforming growth factor-β (TGF-β), nuclear factor of activated T-cells (NF-AT) and insulin/IGF signalling (reviewed in Frame and Cohen, 2001) [2]. This wide-ranging activity has been associated with a broad spectrum of human diseases, such as diabetes, inflammation, neurological disorders and cancer. As a result, GSK-3 inhibitors are being explored for a variety of therapeutic uses (reviewed in Jope, R. *et al.* 2006) [3]. However, in humans, GSK-3 is expressed from two paralogous genes: GSK-3α and GSK-3β. Although available inhibitors act on both proteins, few studies distinguish between GSK-3α and GSK-3β. In fact, many studies entirely neglect GSK-3α, despite its widespread expression and activity. Here, we describe new genetic tools useful for assessing the similarities and differences in the mammalian GSK-3 genes.

GSK-3 is well conserved throughout evolution. To date, homologues have been isolated from all eukaryotes investigated, including the fruitfly (*Drosophila melanogaster*), chickpea *(Cicer arietinum)*, tunicates *(Ciona intestinalis)* and frog (*Xenopus laevis)* (reviewed by Ali *et al.* 2001) [4]. Ancestral organisms have a single GSK-3 related gene whereas in mammals, GSK-3 is encoded by two related genes, GSK-3α and GSK-3β, resulting in 51 kd and 47 kd proteins, respectively. The kinase domains in GSK-3α and GSK-3β are interchangeable, although GSK-3β also has a minor splice isoform (GSK-3β2) that includes an additional 13 amino acids [5]. These proteins differ from one another outside the catalytic region, with GSK-3α possessing an extended glycine rich N-terminus. Similarity between the c-terminal residues dwindles to 36% [6]. Mouse GSK-3 s are suggested to be functionally redundant in Wnt/β-catenin signalling [7]; however, in other signaling pathways, the relative contribution of each GSK-3 protein is less clear.

Mutation of GSK-3β in the mouse leads to a variety of developmental phenotypes including craniofacial anomalies [8], [9], while GSK-3α mutants are reported to be viable [7]. We have previously reported that GSK-3β knockout mice die at birth with cleft palate, bifid sternum and cranial ossification defects [8]. Overall, the etiology of these defects is still unclear, in part because GSK-3 appears to act as a node in multiple developmental pathways, making it difficult to attribute phenotypes to individual pathways. Surprisingly, loss of GSK-3β is comparatively mild, as animals survive gestation and die after birth, with a complete cleft of the secondary palate [8]. In contrast, loss of both GSK-3 genes leads to a catastrophic failure of development, with embryos arrested prior to implantation [7]. This suggests that throughout embryogenesis, GSK-3α can largely compensate for the absence of GSK-3β.

In this paper we describe two new reporter alleles of GSK-3α and GSK-3β, which complement existing GSK-3 alleles (Table 1). Each of these alleles contains a *lacZ* cassette, allowing us to readily visualize β-galactosidase activity driven by the GSK-3α or GSK-3β promoter while disrupting expression of the endogenous gene. These can be compared to methods such as mRNA *in situ* hybridization and immunohistochemistry. In addition, excision of the *lacZ* cassette from the GSK-3α allele creates a Cre-dependent conditional knockout; thus, this allele can be efficiently adapted for a variety of genetic experiments. Using β-galactosidase activity, we compared the expression of GSK-3α and GSK-3β in the craniofacial skeleton. We also found that GSK-3α mutants are homozygous viable, with normal development of the embryonic skeleton and palate. Finally, we found that loss of a GSK-3α allele exacerbates the GSK-3β mutant phenotype, resulting in gestational lethality.

**Table 1:** Current glycogen synthase kinase-3 (GSK-3) alleles.

MGI allele name	Allele type	Reference
GSK-3α^tm1^.^1Jrw^	Conventional null	MacAulay K *et al.*, 2007 [21]
GSK-3α^tm1Dral^	Knock-in of S9A mutation	McManus EJ *et al.*, 2005 [22]
GSK-3α^tm1Jrw^	Floxed/FRT	MacAulay K *et al.*, 2007 [21]
GSK-3α^tm1^.^1Tac^	Floxed/FRT	Hurtado D *et al.*, 2012 [29]
**GSK-3α^tm1a(EUCOMM)Wtsi^**	**lacZ knock-in reporter,** **“conditional ready floxed” allele**	**Barrell W ** ***et al*** **., 2012, this work**
GSK-3β^tm1^.^1Atak^	Conventional null	Kimura T *et al.*, 2008 [30]
GSK-3β^tm1^.^2Ypc^	Conventional null	He F *et al.*, 2010 [14]
GSK-3β^tm1Grc^	FRB* tagged conditional:null in the absence of drug	Stankunas K *et al.*, 2003 [31]
GSK-3β^tm1Jrw^	Conventional null	Hoeflich KP *et al.*, 2000 [9], [22]
GSK-3β^tm1Dral^	Knock-in of S21A mutation	McManus EJ *et al.*, 2005 [22]
GSK-3β^tm1^.^1Ypc^	Floxed/FRT	He F *et al.*, 2010 [14]
GSK-3β^tm1Atak^	Floxed/FRT	Kimura T *et al.*, 2008 [14], [30]
GSK-3β^tm2Jrw^	Floxed/FRT	Tanabe K *et al.*, 2008 [23]
**GSK-3β^tm1Dgen^**	**lacZ knock-in reporter**	**Barrell W ** ***et al*** **., 2012, this work**

Current available alleles of GSK-3α and GSK-3β, including the molecular lesion and references.

## Materials and Methods

### Ethics Statement

All animal work was approved by the King’s College London Ethical Review Process and was performed in accordance with UK Home Office Project Licence 70/6607.

### Mouse Strains

Gsk3α^tm1a(EUCOMM)Wtsi^ mice were obtained from the Wellcome Trust Sanger Institute (MGI ID:4434136) [10]. To generate the “conditional ready” floxed allele, heterozygous mice were crossed for two generations to the “FLPeR” strain [11], deleting the FRT flanked lacZ/neomycin insert. For simplicity, the parental null allele is referred to throughout this work as GSK-3α^L^ and the floxed allele as GSK-3α^fl^. GSK-3β^tm1Dgen^ mice, referred to as GSK-3β^L^, were obtained from Deltagen (MGI ID:3604596). GSK-3β^tm1Grc^ (GSK-3b^Δ^) conditional null mice, previously described [8], were used to generate the compound GSK-3α/β mutants in Table 2. Gestational ages were determined by observation of vaginal plugs (considered to be e0.5), as well as visual inspection after dissection.

**Table 2:** Distribution of compound mutants.

Genotype	Expected	Observed	Cleft Palate
GSK-3α^+/+^; GSK-3β^+/+^	2.5	1	0/1
GSK-3α^+/L^; GSK-3β^+/+^	2.5	1	0/1
GSK-3α^+/+^; GSK-3β^+/Δ^	5	8	0/8
GSK-3α^+/L^; GSK-3β^+/Δ^	5	5	1/5
GSK-3α^+/+^; GSK-3β^Δ/Δ^	2.5	3 (alive)1 (dead)	3/3N/A
GSK-3α^+/L^; GSK-3β^Δ/Δ^	2.5	1 (dead)	N/A

Distribution of genotypes from GSK-3α^+/L^; GSK-3β^+/Δ^ × GSK-3β^+/Δ^ intercrosses dissected at e17.5, compared to expected frequency. Animals with cleft palates are indicated. Animals found dead prior to dissection were not scored for palatal phenotypes, indicated as N/A.

### Genotyping

DNA preparation and PCR genotyping analyses were carried out according to established protocols [8].

### Wild-type GSK-3α was Amplified with Gsk3αF

ACCCTCCAGTCCTTATCCCC and GSK3αR: GCTACCCAGCCTTTCTTCCC, resulting in a 227 bp band. The GSK-3α^L^ allele was amplified with Gsk3αF: ACCCTCCAGTCCTTATCCCC and CAS_R1_Term: TCGTGGTATCGTTATGCGCC, resulting in an 182 bp amplicon. Genotyping for the “FLPed” GSK-3α^fl^ was performed with the same combination of GSK3αF/CAS_R1_Term primers above, resulting in a 182 bp band as well as two additional bands of approximately 400 and 500 bp. Deletion of the original lacZ/neomycin insert was further confirmed by loss of PCR amplification of lacZ.

The GSK-3β^lacZ^ allele was genotyped as follows. Wild-type allele was amplified with GSK3βF: GCAAGGTAACCACAGTAGTGGCAAC and GSK3βR: AGGGATATGGTTCGGTAGTTAAGAG, 263 bp. The mutant allele was detected by PCR for the neomycin resistance gene, GSK3βNeoF: CCCAACATAAGTATGTCTCCC and GSK3βNeoR: ATGCTCCAGACTGCCTT, approximately 600 bp; or, amplification of lacZ, galp1: TTTACAACGTCGTGACTG and galp2: TGATTTGTGTAGTCGGTT. The GSK-3β^Δ^ allele was genotyped as previously described [8].

### Western Blotting

Western blotting and protein preparations were carried out according to established protocols. Antibodies used were: GSK-3α and β (dilution 1∶1000) - Santa Cruz Biotechnology (catalog no. SC-7291), HSP90 (dilution 1∶10000) - Santa Cruz Biotechnology (Catalog no. sc-13119).

### β-galactosidase (*lacZ)* Activity

X-gal staining of β-galactosidase activity was performed as previously described [12]. All lacZ expression was analyzed using heterozygous mutants for GSK-3α or GSK-3β. In all cases, wildtype littermates were also stained as controls for background staining. E13.5 animals were stained whole. At stages e14.5/e15 the skin was removed prior to staining. In postnatal samples, mandibles were separated from the head and skin was removed from the skull.

Bone/cartilage preparations: Skin and internal organs were removed before staining with Alizarin red (for calcified bone) and Alcian blue (for cartilage). Staining was performed as previously described [8].

## Results

To date, there have been four GSK-3α and nine GSK-3β targeted mutations reported (Table 1); but none of these alleles provide a quick and easy method of assessing expression. While mRNA *in situ* hybridization is more straightforward, there have been surprisingly few such analyses of GSK-3α and GSK-3β in the literature and frequently, the assumption is that both genes are ubiquitous. However, several groups have recently reported tissue specific expression of GSK-3s [13], [14]. In the mouse, the most thorough analysis has been in the palate [14]. Immunohistochemical analyses are more common; however, many studies focus on specific cell types and do not distinguish between developmental roles of the two genes. Here, we describe *lacZ* reporter alleles for both GSK-3α and GSK-3β, which are also useful as null alleles. In addition, we describe the skeletal and craniofacial phenotypes of GSK-3α mutant animals, which have not previously been documented.

### Versatile New GSK-3 Alleles

**Figure 1:**
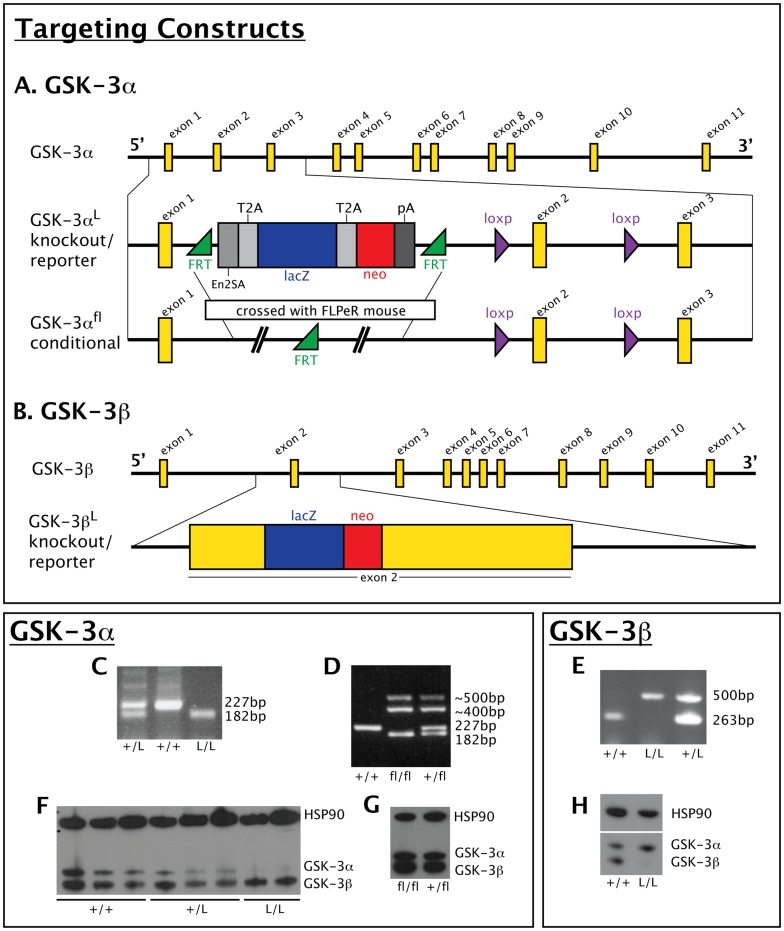
Targeted mutations in GSK-3α and GSK-3β. A. Top row: schematic of mouse GSK-3α locus. Middle row: region containing exons 1–3 are depicted showing cartoon of GSK-3α^L^ allele. Bottom row: schematic depicting genomic locus after crossing with “FLPeR” mice, deleting the FRT flanked region between exon 1 and 2. Maps adapted from http://www.knockoutmouse.org/martsearch/project/27450. Not to scale. B. Top row: schematic of mouse GSK-3β locus. Second row: cartoon of lacZ/neo cassette inserted into exon 2. Not to scale. Abbreviations: FRT = flip recombinase target; En2 SA = En2 splice acceptor; T2A = T2A oligopeptide for ribosomal skipping; pA = polyadenylation; neo = neomycin resistance gene C. Genotyping of GSK-3α^LacZ^ allele from heterozygous (+/L), wildtype (+/+) and mutant (L/L) animals. PCR products: wildtype band (227 bp), mutant band (182 bp). D. Genotyping of GSK-3α^Flox^ allele from wildtype (+/+), homozygous (fl/fl) and heterozygous (+/fl) animals. PCR products: wildtype (227 bp), mutant bands (182 bp with two accessory bands at ∼400 bp and ∼500 bp E. Genotyping of GSK-3β^LacZ^ allele from wildtype (+/+), homozygous (L/L) and heterozygous (+/L) animals. PCR products: wildtype band (263 bp), mutant band (500 bp). F. Western blot analysis of e17.5 kidneys. Genotypes are indicated below. Note expression of both GSK-3α and GSK-3β proteins in wildtype (+/+) animals. Heterozygous (+/L) animals have decreased expression of GSK-3α while homozygous mutants (L/L) samples express no GSK-3α protein. HSP90 was used as a loading control. G. Western blot analysis of adult brains from GSK-3α^fl/fl^ mice show normal expression of GSK-3α and GSK-3β, compared to heterozygous GSK-3α^+/fl^ mice, confirming return of protein expression after intercross with FLPeR mice. HSP90 serves as a loading control. H. Western blot analysis of e13.5 brains show loss of GSK-3β protein in GSK-3β mutant (L/L) animals, compared to wildtype (+/+). HSP90 serves as a loading control.

The GSK-3β mutant mouse was produced by the Wellcome Trust Sanger Institute (WTSI). The targeting construct contains a lacZ/neo cassette inserted between exons 1 and 2 (schematic in Figure 1A) [10]. This cassette is flanked by Flippase Recognition Target (FRT) sites, which allow excision of the lacZ/neo cassette in the presence of Flp recombinase [11]. The targeting construct also introduced two loxP sites surrounding exon 2 of GSK-3β, which provides the potential to create Cre-dependent tissue specific knockouts [15] (Figure 1A). Mutant embryonic stem cells were then generated by homologous recombination. Full details are available from the WTSI website http://www.sanger.ac.uk/mouseportal/). We also describe a GSK-3β^lacZ^ knockout mouse: in this case, the lacZ/neo cassette simply disrupts exon 2 and is predicted to disrupt both splice isoforms (Deltagen, Figure 1B).

The GSK-3α parental allele (GSK-3α^L^) was verified by genotyping of DNA samples from heterozygous, wildtype and mutant littermates (α^L/+^, α^+/+^ and α^L/L^ respectively, Figure 1C). We also intercrossed these mice with the “FlpeR” mice [11], which resulted in removal of the lacZ/neo cassette. Excision of the lacZ/neo cassette was confirmed by PCR analysis (α^fl/fl^, Figure 1D). Genetic deletion of the GSK-3β^lacZ^ allele was confirmed by PCR (bL^+/+^, bL^L/L^ and b^+/L^, Figure 1E).

We next assessed levels of protein expression in our mutants. Western blots showed no detectable GSK-3α protein in extracts from homozygous GSK-3α^L/L^ mutant mice, confirming that the GSK-3α^L^ allele is a complete null (Figure 1F). Note the dose-dependent decrease in GSK-3α protein from wildtype (2 copies), heterozygous (1 copy) and null animals (no copies) (Figure 1F). We also demonstrated restoration of normal GSK-3α protein levels in the “Flped” mice (α^fl/fl^, Figure 1G). Finally, we confirmed that the GSK-3β^L^ allele is also a null (Figure 1H).

### GSK-3α and GSK-3β Reporter Activity in the Palate

**Figure 2:**
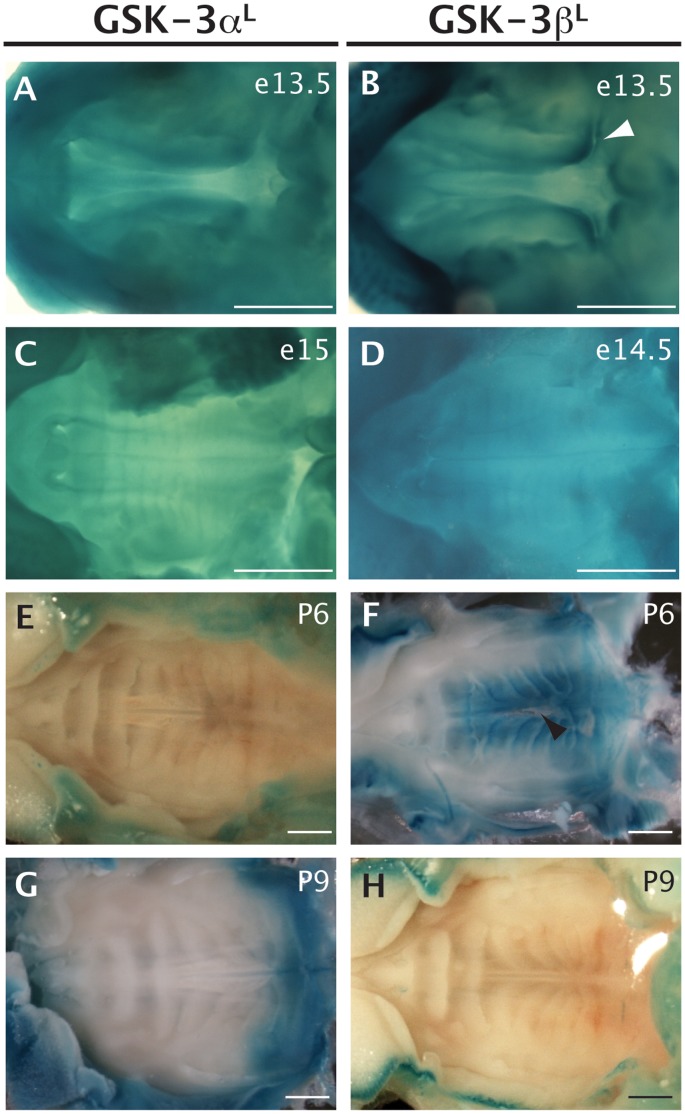
GSK-3α and GSK-3β *lacZ* reporter expression in the palate was visualized by X-gal staining (blue). Orientation: Ventral views of the palatal, anterior to the left, posterior to the right. Scale bar = 1 mm. A, C, E and G. β-galactosidase activity in heterozygous GSK-3α^L/+^ animals. B, D, F and H. β-galactosidase activity in heterozygous GSK-3β^L/+^ animals. A–B. At e13.5, both reporters are expressed. In the GSK-3β reporter mice, there is enriched expression in the posterior palatal shelves (white arrowhead) that is not evident in the GSK-3α animals. C–D. At e14.5/15 there are similar expression levels in both lines. E. At postnatal day 6 (P6), there is no palatal expression in the GSK-3α reporter. F. At postnatal day 6 (P6), midline expression of the GSK-3β reporter persists in a defined region adjacent to the midline seam. This animal also displays a small cleft (black arrowhead). G–H. By P9, no expression of is seen in either reporter mouse, except for a small domain in the posterior palate, GSK-3α (G).

In the mouse, the palatal shelves are evident at e11.5, arising from the oral aspect of the maxillary process. By e13.5 the palatal shelves have grown vertically. At ∼e14 the shelves elevate, resulting in the two shelves abutting by ∼e14.5/15 [16]. Clefting of the palate can occur following failure of one of these steps or if the tongue physically impedes elevation [16]. Because GSK-3β mutants have a cleft palate resulting from defects in palatal morphogenesis at e13.5 [8], [14], [17], we examined expression of both GSK-3α and GSK-3β at this stage. At e13.5, we found robust β-galactosidase activity in all the palatal shelves (Figure 2A and 2B), confirming a previous report from He *et al.* 2000 [14], showing that both GSK-3α and GSK-3β mRNA are strongly expressed in the palatal epithelium at these stages. We also note a posterior enrichment of *lacZ* staining in the GSK-3β knock-ins (Figure 2B, arrowheads). By e14.5/e15, when the shelves are abutting, expression from both loci has become uniform (Figure 2C and 2D).

We then examined postnatal expression of both GSK-3 genes in the palate. At P6 we see no expression from the GSK-3α promoter in the palate (Figure 2E). In contrast, β-galactosidase activity was detected in the GSK-3β knock-in, extending out from the midline (Figure 2F). In addition, we occasionally noted a partial cleft in the GSK-3β^L/+^ animal. By P9 there is minimal β-gal activity in the palate of either mouse line (Figure 2E and 2F), although some expression from the GSK-3α locus appears posteriorly, at the junction of the hard and soft palate (Figure 2E).

### GSK-3α and GSK-3β Reporters in the Calvaria

**Figure 3:**
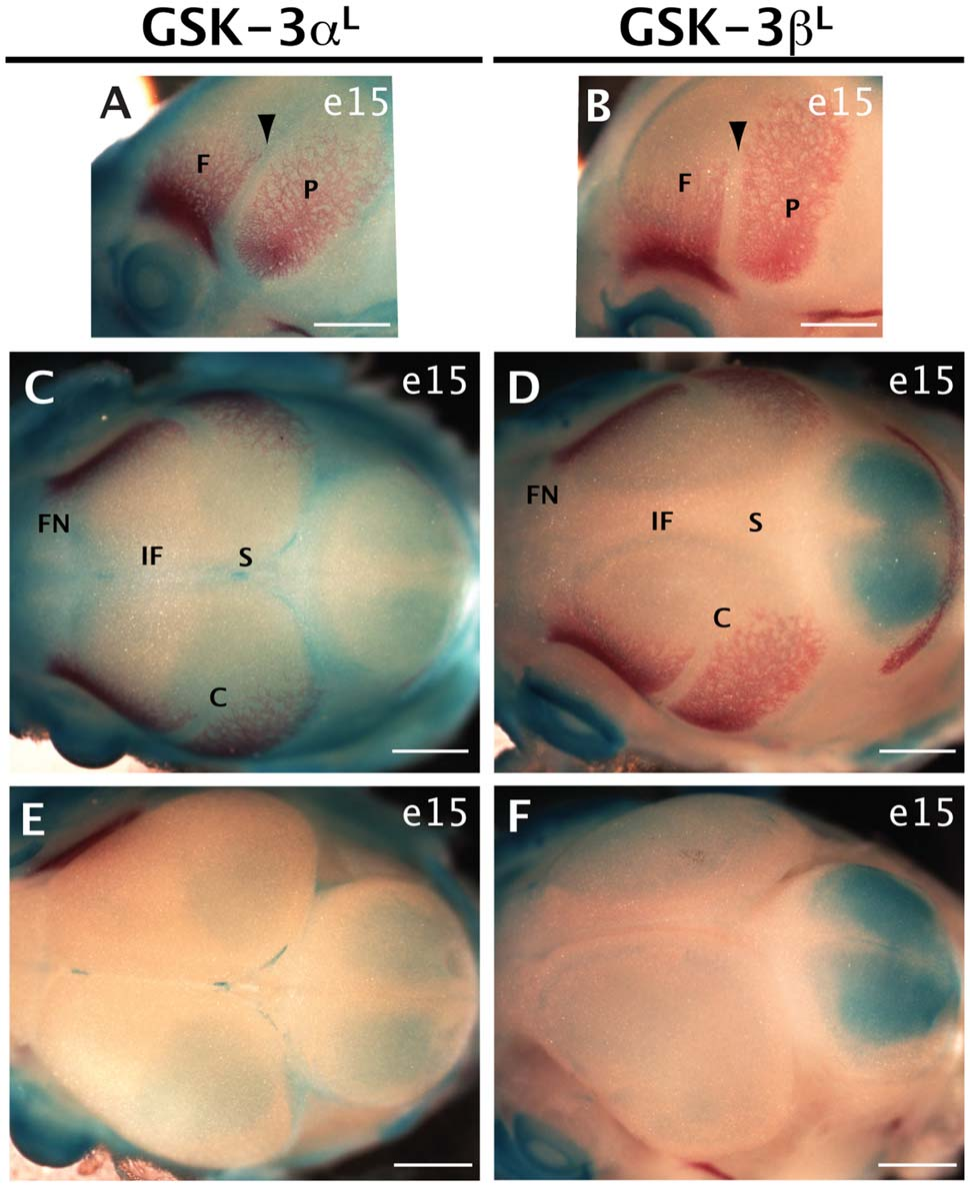
GSK-3α and GSK-3β *lacZ* reporter expression in the embryonic (e15) skull vault was visualized by X-gal staining (blue). A–D. Skulls were counterstained with Alizarin red, to mark the developing calvarial bones. Scale bar = 1 mm. A–B. Lateral views of the coronal suture (arrowhead), with frontal bone (F) on the left and parietal bone (P) on the right. Expression of the GSK-3α reporter can be seen between the two bones (A, arrowhead) whereas GSK-3β expression is not evident (B, arrowhead). C–D. Dorsal view of the skull vault showing the frontonasal (FN), interfrontal (IF), sagittal (SS) and coronal (CS) suture regions. There is fairly generalised expression of GSK-3α in these suture regions (C) whereas this is not seen with GSK-3β (D). Specific areas of skull staining are easier to visualize when left intact. E–F. Skull vaults have been removed, revealing GSK-3 reporter expression in the brain. Note, because of penetration issues, this staining is not a true reflection of total reporter levels in the brain. GSK-3α expression is present in the posterior cerebral cortex and in the midbrain (E). GSK-3β expression is seen in the cerebral cortex as well, with additional expression in the midbrain (F). Compare (C–D) to (E–F) respectively to appreciate skull specific staining in (C–D).

The majority of the skull vault, or calvaria, is made of paired flat bones that meet at fibrous joints called sutures. These sutures provide a dynamic osteogenic front for the skull bones. In addition, sutures act as stress absorbers while allowing the cranium to deform during birth [18]. Previous work suggests that GSK-3 is important during calvarial development [8]. Therefore, we examined expression of β-gal reporter activity in the skull, at two time points: e15, as the skull bones are developing, and postnatally, after the bones have come together. To distinguish between reporter activity in the skull versus the underlying brain, we also visualized the calvarial bones by staining with the Alizarin Red (Figures 3A–D). We then dissected the calvaria away from the brain, further revealing β-gal activity localized to the neural tissues underneath (Figures 3E–F).

At e15, the GSK-3α reporter is active in the coronal suture (Figure 3A, arrowhead) but no expression was found from the GSK-3β reporter (Figure 3B). We also noted that GSK-3α reporter activity was found in the majority of cranial sutures (Figures 3C) whereas there was minimal GSK-3β reporter activity in the sutures (Figure 3D). Upon removal of the calvaria, it was clear that the to a large extent, the reporter activity seen was localized to the underlying neural tissues, particularly in the case of GSK-3β (compare Figures 3C–D to Figures 3E–F).

**Figure 4:**
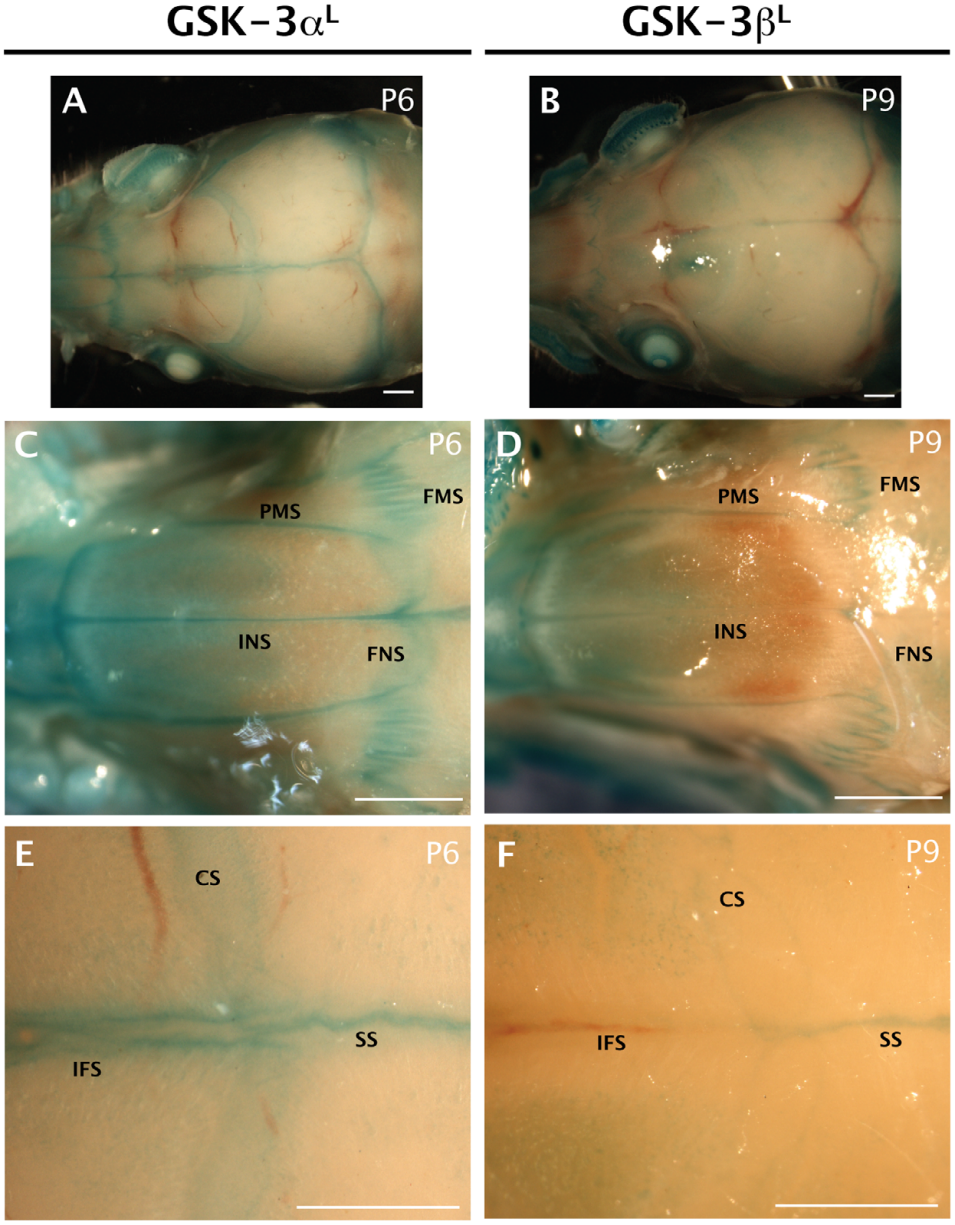
GSK-3α and GSK-3β *lacZ* reporter expression in the postnatal skull vault was visualized by X-gal staining (blue). Scale bar = 1 mm. A–B. Dorsal view of GSK-3α and GSK-3β reporter expression in postnatal sutures (P6 and P9, respectively). Robust GSK-3α reporter expression is seen in the sutures compared to minimal GSK-3β reporter activity. C–D. Nasal suture region. Both GSK-3α and β expression is seen in the frontonasal (FNS) and frontomaxillary (FMS) suture. Expression of the GSK-3α reporter is also found in the internasal (INS) and premaxillary-maxillary (PMS) suture (C). GSK-3β is also expressed in the premaxillary-maxillary suture but only in the anterior internasal (INS) suture. E–F. Cranial suture region. GSK-3α is expressed in the interfrontal (IFS), coronal (CS) and sagittal (SS) sutures (E); but, GSK-3β is only expressed in the sagittal suture (F).

Postnatally, we observed that the GSK-3α reporter is again expressed in all sutures (Figure 4A) while GSK-3β promoter activity is minimal (Figure 4B). In the anterior region of the skull (nasal region) we observed GSK-3α reporter expression in all the sutures (Figure 4C) whereas GSK-3β is only expressed in the premaxillary-maxillary suture (PMS), the anterior internasal suture (INS) and the frontomaxillary suture (FMS) (Figure 4D). We see a similar expression pattern at the intersection between the interfrontal (IFS), sagittal (SS) and coronal sutures (CS), with GSK-3α^lacZ^ expressed in all 3 sutures and GSK-3β^lacZ^ only detectable in the sagittal suture (Figure 4E–F).

In both cases, staining of the skulls was kept to a minimum, in order to visualize specific differences in expression. When we allow further staining to occur, both GSK-3α and GSK-3β appear ubiquitous (data not shown).

### GSK-3α Mutants have Normal Development of the Skeleton

**Figure 5:**
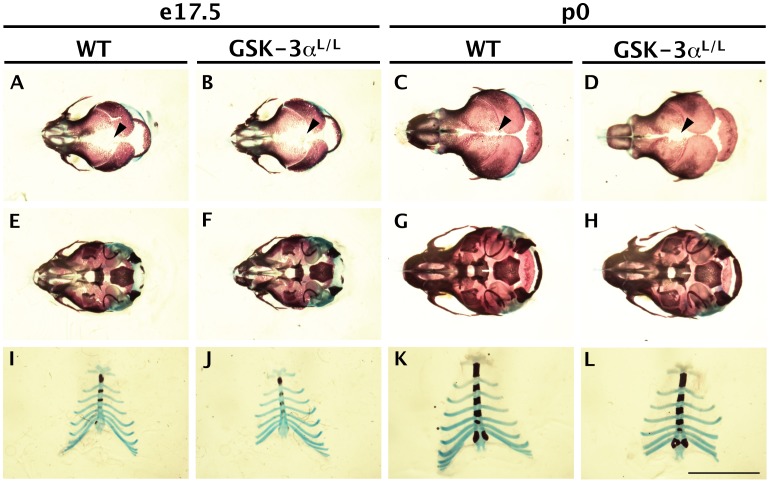
Skeletal preparations of wildtype GSK-3α+/+ and mutant GSK-3αL/L. Alizarin red staining of bone and alcian blue staining of cartilage. Scale bar = 5 mm. A-D. Bone and cartilage staining of skull vaults. At e17.5 (A–B), wildtype and mutant animals looked quite similar. Anterior fontanelle is marked with black arrowhead. At p0, mutant GSK-3α^L/L^ animals had a somewhat wider anterior fontanelle (D, black arrowhead). E-H. Bone and cartilage staining of cranial base and other associated skeletal elements. (Skull vaults have been removed.) Wildtype and mutant littermates looked identical at both stages. I–J. Bone and cartilage staining of sternum and ribs. Wildtype and mutant littermates looked similar at both stages.

We have shown previously that all GSK-3β knockouts die at birth with cleft palates and skeletal defects [8]. Although GSK-3α mutants are viable, it has not been reported if the GSK-3α null mutants have any skeletal defects, and it is conceivable that skeletal anomalies have been overlooked. Therefore we examined GSK-3α^−/−^ mice for skeletal malformations by performing bone/cartilage preparations at e17.5 and P0 (Figure 5). We focused on the skull vault, the cranial base and the sternum as these are areas that are affected in the GSK-3β mutants [8].

We found that the GSK-3α mutants did not show any obvious phenotypes relating to these structures (Figure 5). At e17.5 and P0 there were no discernible malformations in the skull vault or cranial base, and there was no sternal cleft or bifid sternum (Figure 5I–L). Also, there were no other obvious skeletal deformities present in the GSK-3α mutants. The interfrontal sutures of the e17.5 and P0 GSK-3α^L/L^ (Figure 5B and 5D, arrowheads) were subtly wider compared to wildtype littermate control (Figure 5A and 5C). However, this could be due to normal developmental variation amongst the litter. During dissection, we also confirmed that these animals had normal closure of the palate (data not shown). Finally, we confirmed that GSK-3α^L/L^ mice live to adulthood and can breed (data not shown).

### Loss of One Copy of GSK-3α may Exacerbate the GSK-3β Phenotype

Previous studies have shown that loss of both GSK-3 genes is catastrophic, leading to preimplantation lethality [7]. Indeed, we were unable to find any GSK-3 double homozygous mutants. We then set out to determine what phenotypes, if any, resulted from the loss of three alleles of GSK-3 (GSK-3α^+/L^; GSK-3β^Δ/Δ^). To do this, we intercrossed GSK-3α^+/L^; GSK-3β^+/Δ^ mice with GSK-3β^+/Δ^ mice. Examination of two litters at e17.5 showed non-Mendelian ratios (Table 2), suggesting that some animals were dying prior to this stage. We also observed some variance regarding palatal clefting. As expected, clefts were seen in animals homozygous for GSK-3β; however, we also saw a cleft in one (of five) GSK-3α^+/L^; GSK-3β^+/Δ^ mice (Table 2). These data suggest requirements for both GSK-3s during embryonic development. Future studies will be needed to clarify the relative contributions of each gene.

## Discussion

In mammals, there are two GSK-3 genes encoding GSK-3α, GSK-3β and a less-studied splice isoform GSK-3β2 that appear to have overlapping activities and target specificities [5], [6]. Current evidence linking GSK-3 to a variety of human disorders has led to the exploration of dozens of pharmacological inhibitors of GSK-3. All of these inhibitors bind in the ATP-binding pocket of GSK-3 and cannot differentiate between GSK-3α and GSK-3β [19]. Because GSK-3 inhibition is likely to have broad pleiotropic effects, further studies distinguishing *in vivo* roles for GSK-3α and GSK-3β will be necessary for better targeting of GSK-3 function.

Recently, it has become clear that some of the functional divergence in GSK-3 proteins can be attributed to tissue specific interactions. For example, the majority of glucose/glycogen homeostasis appears to depend mainly on GSK-3α, with a minor contribution of GSK-3β in skeletal muscle [20]–[22]. However, loss of GSK-3β alone can rescue a mouse model of diabetes [23]. Even more striking, in the heart, GSK-3β is suggested to be important for embryonic cardiomyocyte development [24], while GSK-3α is implicated in postnatal stress responses via β-adrenergic signalling [25]. Despite these data, comparatively little is known about the relative transcriptional regulation of these two genes. In fact, the majority of reports suggest that both genes are expressed in most cell types (reviewed in Doble & Woodgett, 2003 [26]).

In this paper we have outlined two new reporter alleles for GSK-3. These alleles enable us to comparatively assess expression of GSK-3α and GSK-3β, as well as allowing us to determine areas of transcriptional upregulation at different time points. Because of the known GSK-3β phenotypes, and because mRNA expression in the palate is well documented, we focused specifically on the palate and the cranial sutures. Our data suggest that there is indeed differential expression of GSK-3α compared to GSK-3β, which would account for some of the loss of function phenotypes.

GSK-3β is known to be required for proper growth and elevation of the palatal shelves. Consistent with this, we found activity of the GSK-3β reporter during critical developmental periods, with enriched expression in the posterior palate at e13.5. GSK-3α does not show this increased expression at e13.5 raising the possibility that GSK-3β is the key player during embryonic palatal development (Figure 2). This notion is further supported by the observation that GSK-3α mutants have normal palate development. Furthermore, the GSK-3β reporter continues to be expressed in the midline of the palate at P6, while GSK-3α expression is absent (Figure 2). We have previously shown that GSK-3β is required during ossification of the palatine bone [17], this role might continue postnatally, perhaps during fusion of the palatine suture.

During development of the cranial sutures, we were surprised to find robust expression of the GSK-3α reporter and more refined expression of the GSK-3β reporter (Figures 3–4). Both GSK-3α and GSK-3β mutants display some cranial phenotypes (Figure 5 and [8]); however, it may be that GSK-3β phenotypes can be attributed to earlier embryonic effects, prior to ossification (HL Szabo-Rogers and KJ Liu, *in preparation).* This would suggest that GSK-3α will prove to be more important during postnatal sutural maintenance. Certainly, GSK-3α nulls do not exhibit any of the severe embryonic craniofacial and skeletal phenotypes seen in GSK-3β mutants [8]. However, the subtle patency of the interfrontal suture supports the idea that GSK-3α is important in sutural development (Figure 5). Long-term studies will be needed to address this possibility.

In the future, it will be intriguing to examine GSK-3 expression in tissues where the individual genes play critical roles, such as the heart and cartilage [24], [25], [27], [28]. While there are more traditional methods for assessing mRNA and protein expression levels, surprisingly little analysis has been performed on GSK-3α and GSK-3β, even in the papers that produced the first knockouts [6], [7], [9]. Differing expression patterns have been reported in human skeletal muscle, analyzed by western blotting [13], and in the mouse palate [14]. However, even though distinct roles are reported in systems such as the heart, GSK-3 proteins are assumed to have overlapping expression patterns [24], [25], [28]. The generation of these mice were aimed in part to provide more detailed expression analysis; selected data generated by WTSI and Deltagen are already available on public websites, as follows:

GSK-3α (Wellcome Trust Sanger Institute): http://www.sanger.ac.uk/mouseportal/phenotyping/MCCU/embryo-lac-z-expression/http://www.sanger.ac.uk/mouseportal/phenotyping/MCCU/adult-lac-z-expression/

GSK-3β (Deltagen): http://www.informatics.jax.org/external/ko/deltagen/3228.html/).

There are of course some caveats to using lacZ reporters in the mouse, including background staining, problems with tissue penetration and difficulties comparing reporter strains. Nevertheless, these new alleles are able to give us some insight into overlapping and non-overlapping domains of GSK-3α and GSK-3β expression. Furthermore, because these are genetic knockins, they could be combined with other mutant alleles, such as the non-inhibitable (S9A/S21A) GSK-3 mutants [22], to test for feedback regulation of either protein. In particular, Gillespie *et al.*, 2003 [27], report that deletion of GSK-3β in chondrocytes leads to compensatory upregulation of GSK-3α [27]. It is unclear whether this occurs via increased transcription of GSK-3α, or via post-transcriptional means. Our animals would be useful tools to distinguish between these possibilities. Alternatively, these reporter alleles could also be used to search for factors that can differentially activate GSK-3 transcription; very little is known about this level of regulation. In summary we describe two new genetic tools to assess spatial and temporal expression of GSK-3. Together, these new mice provide us with a quick and easy approach to compare expression patterns, as well as two multipurpose genetic alleles for mutant analyses.
